# The involving progress of MSCs based therapy in atherosclerosis

**DOI:** 10.1186/s13287-020-01728-1

**Published:** 2020-06-05

**Authors:** Ying Lin, Wei Zhu, Xiaomin Chen

**Affiliations:** 1 School of Medicine, Ningbo University, Ningbo, Zhejiang China; 2 Department of Cardiology, Ningbo First hospital, Ningbo, Zhejiang China; 3 Department of Cardiology and Key Lab of Cardiovascular Disease, Second Affiliated Hospital of Zhejiang University School of Medicine, Hangzhou, China

**Keywords:** Atherosclerosis, Mesenchymal stem cell, Immunomodulation

## Abstract

Atherosclerosis is a chronic progressive vascular inflammation characterized by lipid deposition and plaque formation, for which vascular cell dysfunction and impaired immune responses are involved. Up to now, lipid-lowering drugs remain the main therapy for treating atherosclerosis; however, the surgical or interventional therapy is often applied, and yet, morbidity and mortality of such cardiovascular disease remain high worldwide. Over the past decades, an anti-inflammatory approach has become an important therapeutic target for dealing with atherosclerosis, as altered immune responses have been regarded as an essential player in the pathological process of vascular abnormality induced by hyperlipidemia. Interestingly, mesenchymal stem cells, one type of stem cells with the capabilities of self-renewal and multi-potential, have demonstrated their unique immunomodulatory function in the various pathological process, especially in atherosclerosis. While some controversies remain regarding their therapeutic efficacy and working mechanisms, our present review aims to summarize the current research progress on stem cell-based therapy, focusing on its immunomodulatory effects on the pathogenesis of atherosclerosis and how endothelial cells, smooth muscle cells, and other immune cells are regulated by MSC-based therapy.

## Background

Atherosclerosis (AS) is now being regarded as an aging-associated chronic vascular inflammatory disease, which also involves the inflammatory and immune responses. Studies have shown that vascular endothelial cells (ECs), smooth muscle cells (SMCs), and immune cells have participated in and contributed to the atheroma formation, progression, and rupture at each stage of the atherosclerosis. While preventing the development or worsening of atherosclerotic lesion, such as the use of hyperlipidemia-lowering drugs, remains the major therapeutic strategy, which has significantly improved the prognosis of cardiovascular diseases, however, for patients at high risk or with detectable advanced plaque lesion, the progress of atherosclerotic plaque still has a high mortality rate [1]. Thus, it is still highly desirable to seek solutions for inhibiting the progression of the existing plaque or promoting the formation of stable lesions.

In addition to its inherent biological properties of stem cells, such as multi-potential differentiation and self-renewal capabilities, MSCs have become a hotspot in various research fields with their characteristics of rich sources, easy culture, and expansion [2]. MSCs have been shown to be able to exert their strong anti-inflammatory and immunomodulatory effects in the various pathological process of cardiovascular diseases, especially in the setting of myocardial infarction (MI) and AS [3]. However, there remain some unsolved issues and challenges regarding MSC-based therapy when applied to AS, including the dosage, i.e., the number of the MSCs for each transplantation, their therapeutic efficacy, and the potential side effects on the recipient receiving allogeneic MSCs. Nevertheless, there are some studies demonstrating that MSC therapy can lead to regression of plaque via regulating the functions of ECs, vascular smooth muscle cells (VSMCs), and immune cells. Of note, bone marrow has been a most widely applied source in the MSC-based therapy within those various sources, which is mainly discussed in our review. Therefore, it is important to review all these data and discuss the detailed information about the therapeutic effects and their efficacy when bone marrow-derived MSCs (BMSCs) are applied to treat patients with AS, providing insights into the MSC-based therapy in the area of AS and vascular biology.

## Anti-inflammatory and immunomodulating effects of BMSCs

### Pro- vs. anti-inflammatory modulation via paracrine effects of BMSCs

Early in 1960s, the transplantation of stem cells has been postulated to have a potential regenerative capability, which can be applied to treat the patients with cardiovascular diseases, especially for generating cardiomyocytes to compensate those lost in the event of MI [4]. However, recent studies showed that fewer than 1% of the transplanted cells actually reached the target tissues with most of the transplanted cells being trapped in the liver, spleen, and lung [5]. In fact, even the transplanted BMSCs that homed and engrafted into the infarct area do not exhibit any evidence demonstrating that they have differentiated into new cardiomyocytes [6]. Indeed, in the setting of MI, BMSC-based therapy reduces the levels of pro-inflammatory factors such as interleukin-1 (IL-1), interleukin-6 (IL-6), and tumor necrosis factor alpha (TNF-α), while it increases the levels of anti-inflammatory factors such as IL-10 and TGF-β, which are associated with higher expression levels of vascular endothelial growth factor (VEGF) and hepatocyte growth factor (HGF), and all of these eventually improve inflammatory-immune responses and promote the survival of cardiomyocytes, hence preventing the adverse remodeling process [7]. Further study has shown that injection of BMSC-conditioned supernatant alone has achieved similar effects as the BMSC transplantation [8], strongly supporting that it is the paracrine effects of BMSCs that modulate the progression and the pathological remodeling. Of note, a substantial amount of biological active substances secreted by BMSCs are enclosed within extracellular vesicles (EVs) that contain various growth factors and chemokines.

### Immuno- and inflammatory modulation by BMSCs therapy in reverse cardiac remodeling

While BMSCs show their therapeutic effects on cardiac remodeling process through paracrine effects, studies have also indicated that immunomodulatory effects can also show their protective effects [9–14], such as modulating the function of regulatory T cells (Tregs) and reducing the proliferation of T cells. T cells, a central player in the adaptive immune system, take an important role in affecting acute MI and cardiac remodeling via their proliferation and differentiation [15]. Several in vitro experiments have shown that BMSCs can inhibit the proliferation of T cells via disturbing the cell cycle [9], thereby the anti-proliferative effect of BMSCs causes a shift in T cell differentiation from a pro-inflammatory state (Th1, Th2, Th17etc.), with decreased interferon-gamma (IFN-γ) production, to an anti-inflammatory state with an increase in interleukin 10 (IL-10) production by Tregs [10]. Moreover, BMSCs release anti-inflammatory cytokines to affect the macrophage M2 polarization in MI [11]. Of note, inflammation resolution is an important phase for reducing scar formation and protecting cardiac function after MI [16]. Tregs exert their strong immunosuppressive function in the stage of inflammation resolution in post-ischemic cardiac remodeling via enhancing tolerance induction and promoting macrophage phenotypic transition from a pro-inflammatory state (M1) to an anti-inflammatory state (M2) [16]. BMSC-mediated immunosuppression partially relies on the soluble immunosuppressive factors secreted by BMSCs including HGF, transforming growth factor beta (TGF-β), IFN-γ, nitric oxide (NO), interleukin-2 (IL-2), prostaglandin E2 (PGE2), indoleamine-2,3-dioxygenase (IDO), and IL-10 [12, 13], further regulated by the secreted EVs. It has been well-established that various mRNA, microRNA, and non-protein encoding RNA contained in BMSC-EVs are delivered to recipient cells and affect intracellular immunological responses, which are involved in processes of antigen presentation, angiogenesis, coagulation, and programmed cell death [14]. However, limited data has showed BMSCs immunomodulation on Tregs proliferation and differentiation in vivo. Intracardiac injection of BMSCs in patients with chronic ischemic heart failure was found to have a significant improvement in both reducing scar tissue and increasing myocardial function, but without improvement in cardiac function [17–19]. Although the roles of BMSCs in cardiac remodeling including anti-apoptosis and pro-survival of ischemic myocytes [20], promoting endothelial progenitor cell proliferation [21], increasing the number of M2 macrophages [22], and alleviating local and systemic inflammation have been reported in several literatures, the effect of BMSCs on mediating the crosstalk between immune cells and inflammatory cells is still not fully understood. Furthermore, the maximum efficacy for BMSCs treated in cardiac remodeling depends on the optimal injection phase and duration time, all of which can influence the immunomodulation efficacy of BMSC-based therapy [23].

### Immuno- and inflammatory modulation by BMSCs therapy in AS

**Fig. 1:**
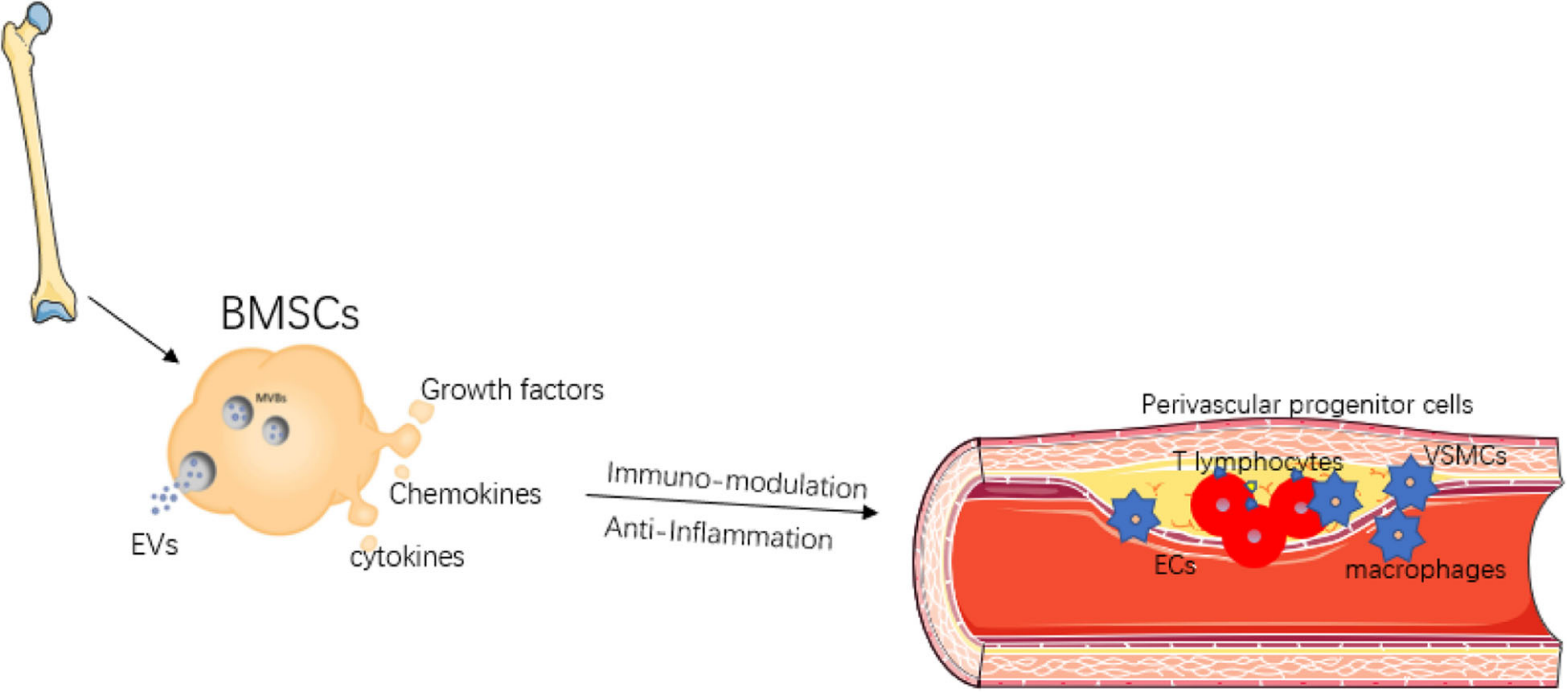
The role of BMSCs in atherosclerotic plaque

As reported, secretome from BMSCs dictates the bioactivity of immune cells, being of either pro- or anti-inflammatory property, which actually orchestrates inflammatory response during progress of cardiovascular disease. Considering AS as a chronic inflammation disease caused by a dysregulated immunity system, BMSC-based therapy might exert protective effects on pathological progression of atherogenesis (Fig. 1). Growing body of evidence has demonstrated that allogenic BMSC transplantation can stabilize the plaque via altering immune cellular components within atherosclerotic plaque to alleviate inflammation [24–28]. Being similar to the immunomodulatory effects during the cardiac remodeling process as described above, in the pathogenesis of atherosclerotic plaque, BMSCs increased the ratio of Tregs over CD4^+^ T cells and promoted the macrophage differentiation towards the M2 phenotype, thereby reducing the monocyte infiltration and inflammatory response [26]. However, BMSCs from patients with AS lost their inherent immunomodulatory capability, instead they excrete a set of pro-inflammatory secretome, being strikingly different from BMSCs derived from healthy donors [29]. A study has validated that EVs contain a spectrum of components such as mRNA, microRNA, non-coding RNA, proteins, and even organelles, such as mitochondria [28]. EVs derived from BMSCs can infuse with recipient cells to regulate gene expression at both transcriptional and post-transcriptional levels of the recipient cells, hence affecting recipient cell function [30]. Interestingly, the immunomodulatory capabilities of BMSCs are not constitutive. It is mandatory that BMSCs immunomodulatory function should be primed and activated under specific circumstances [31]. For instance, for BMSCs under hypoxic treatment, inflammatory stimuli, and other environments, the beneficial effects against AS appeared to be enhanced, showing that an increased number of EVs are released into conditioned medium of BMSCs [27]. Interestingly, BMSCs can also improve the oxidative stress state and energy metabolism of recipient cells by transmitting normal mitochondrial organelles or mitochondrial DNA macrophages [28]. Both the in vivo and in vitro experiments mentioned above have pointed out several interesting questions. For example, where and how do BMSCs exert its immunomodulation on myeloid cells, and how lesioned Tregs are differentiated and recruited from other immune organs especially bone marrow and spleen. However, whether and how BMSCs can influence the action of hematopoietic progenitors in other locations are still not fully known. Since intravenous injection of BMSCs in a model of atherosclerosis as showed above can alleviate the inflammatory circumstance, a question regarding how BMSCs influence the crosstalk between lesion-localized immune cells and local non-inflammatory cells such as ECs and SMCs is therefore put forward for researchers working in this area. Moreover, it is also interesting to answer how BMSCs play its anti-inflammatory and/or immunosuppressive roles in each stage of AS including the initial, later further progression, and advanced plaques, as each stage would have their unique pathologic characteristics. Therefore, we will discuss these issues in more detail, hoping that the thorough discussion on these issues related to the MSC-based therapy will provide some deep insights for better understanding the mechanisms and efficacy of BMSCs transplantation for treating patients with AS.

## Responses of resident vascular wall progenitor cells to AS

### Impaired immunomodulation of resident vascular wall progenitor cells within atherosclerotic plaque

Recent discoveries have uncovered the presence of a range of multipotent and lineage-restricted progenitor cells in the vascular adventitia, sharing high proliferative capacity and potential to re-generate ECs, VSMCs, and hematopoietic or mesenchymal cell progeny [32]. Vascular wall progenitor cells are quiescent in physiological conditions and can be activated by a variety of stimuli such as hyperlipidemia and other inflammatory cytokines or chemokines [33, 34]. Sca1^+^ adventitial progenitor cells showed enhanced capability of proliferation and migration and can differentiate into VSMCs and ECs contributing to the neointima formation in the setting of vascular injury [34]. Using single-cell gene sequencing techniques to compare the adventitial progenitor cells derived from wild-type and ApoE^−/−^deficient mice, research studies have found that several genes related to cell migration and matrix protein degradation displayed a significant alteration. Among them, a group of genes have a role in regulating progenitor stem cell migration; some genes were crucial for endothelial regeneration, and five genes were associated with leukocyte adhesion [35], indicating gene alternation is related to endothelium repairment. Moreover, the activation of adventitial progenitor stem cells depends on the oxidized LDL, cholesterol, and chemokines such as CCL2, CXCL1, and CCR2, all of which are actually released by vascular inflammatory cells and/or immune cells within the plaque [36]. However, MSCs derived from extravascular tissues exert an opposite role in atherogenesis, which showed a diminished capability of immunoregulation owing to an ineffective secretory profile [29].

### The comparison of immunomodulatory capability between BMSCs derived from patients with AS and healthy donors

Compared to allogeneic BMSCs derived from health donors, BMSCs from AS patients have significant differences in paracrine effects, either increasing the pro-atherogenic substances or decreasing athero-protective mediators [37]. In fact, BMSCs isolated from aged and peripheral vascular disease patient characterized as a pathological status of excessive mitochondrial oxidative stress and imbalanced energy metabolism resulting in the presence of chronic inflammation [38, 39]. Moreover, the impaired immunomodulation function of BMSCs can be partially restored by reactive oxygen species (ROS) scavenger and enhanced mitochondrial function in vitro research [40]. Therefore, the differences in their inherent immunomodulation capability of MSCs that are isolated from healthy donors and patients with atherosclerotic lesion, can certainly determine their therapeutic effects on AS, as the healthy BMSCs exhibit anti-athero effects whereas the BMSCs from diseased donors might well be showing their pro-athero effects. Thus, there are many unsolved issues in BMSC-based therapy which certainly need further research work to clarify on. Since progenitor stem cells located in vascular wall have been shown to be athero-protective, searching for changes in gene expression between BMSCs obtained from healthy donors and diseased ones would elucidate the potential molecular targets for rescuing the impaired function of endogenous BMSCs in atherosclerotic patients and re-establish their immune-inflammatory modulating function. This would be a promising treatment for AS. In addition, ongoing research has shown that mitochondria can also be manipulated to enhance autologous BMSC cell function, hence the efficacy of BMSC-based therapy. While BMSC-based therapy still holds great promise for treating patients with AS, there are still many questions that need to be answered. For example, it remains unknown whether there exists potential crosstalk between resident endogenous vascular MSCs and allogenic transplanted BMSCs, and whether transplanted BMSCs can help to enhance the reparative function of those endogenous vascular progenitor stem cells. Answering these questions would certainly not only help to understand the natural biological functions, i.e., the secretome profiles from both endogenous MSCs and transplanted BMSCs, being either pro- or anti-atherosclerotic to atherosclerotic lesion, but also would further elucidate the underlying molecular mechanisms which would provide unique therapeutic targets for treatment of AS in the near future.

## The role of allogeneic BMSCs on ECs

Endothelial cell dysfunction (ECD) is the primary cause of AS. Under the influence of AS high-risk factors such as lipid metabolism disorder and blood flow instability, the integrity of the vascular intima lining is interrupted, then continuous inflammatory stimulation and accumulating lipid deposition lead to the development of ECD. The dysregulated ECs mainly manifest the imbalance of endothelial nitric oxide synthase (eNOS) and NO production, leading to increased intracellular oxidative stress and a series of phenotypic changes in ECs such as inflammation phenotype and foam cell-like phenotype [41]. On the one hand, growing literatures have reported that acute and transient inflammatory stimulation can enhance the capability of BMSCs to improve the ECs function [42]. NO is mainly produced by the eNOS system and participates in vasodilation and protecting the ECs function. In the progression of AS, inactivation of the Akt/eNOS pathway leads to the degradation of eNOS, causing the reduction of NO production which increases reactive oxygen species (ROS) generation by ECs and results in intracellular oxidative stress and the development of AS. It was found that with co-culture of BMSCs with ECs that were stimulated with ox-LDL, BMSCs can activate the eNOS system by increasing the expression of interleukin-8 (IL-8) and macrophage inflammatory protein-2 (MIP-2) to promote the production of NO, and then improve the function of ECs [43]. Meanwhile, BMSCs can also activate β-catenin-mediated Wnt signaling pathway by secreting Wnts protein to reduce ECs apoptosis via reducing oxidative stress [44]. TNF-α-stimulated ECs up-expressed cell adhesion molecules (CAMs), a factor that can promote monocyte/macrophage recruitment and adhesion, to initiate the occurrence of AS [44]. It has been shown that when ECs were co-cultured with conditional supernatant or purified exosomes derived from BMSCs, HGF within the exosomes can inhibit CAM production by ECs via downregulating mitogen-activated protein kinase (MAPK) and nuclear factor kappa-B (NF-κB) pathways, thereby reducing the recruitment of macrophages [24].

## The role of allogenic BMSCs on EPCs

**Fig. 2:**
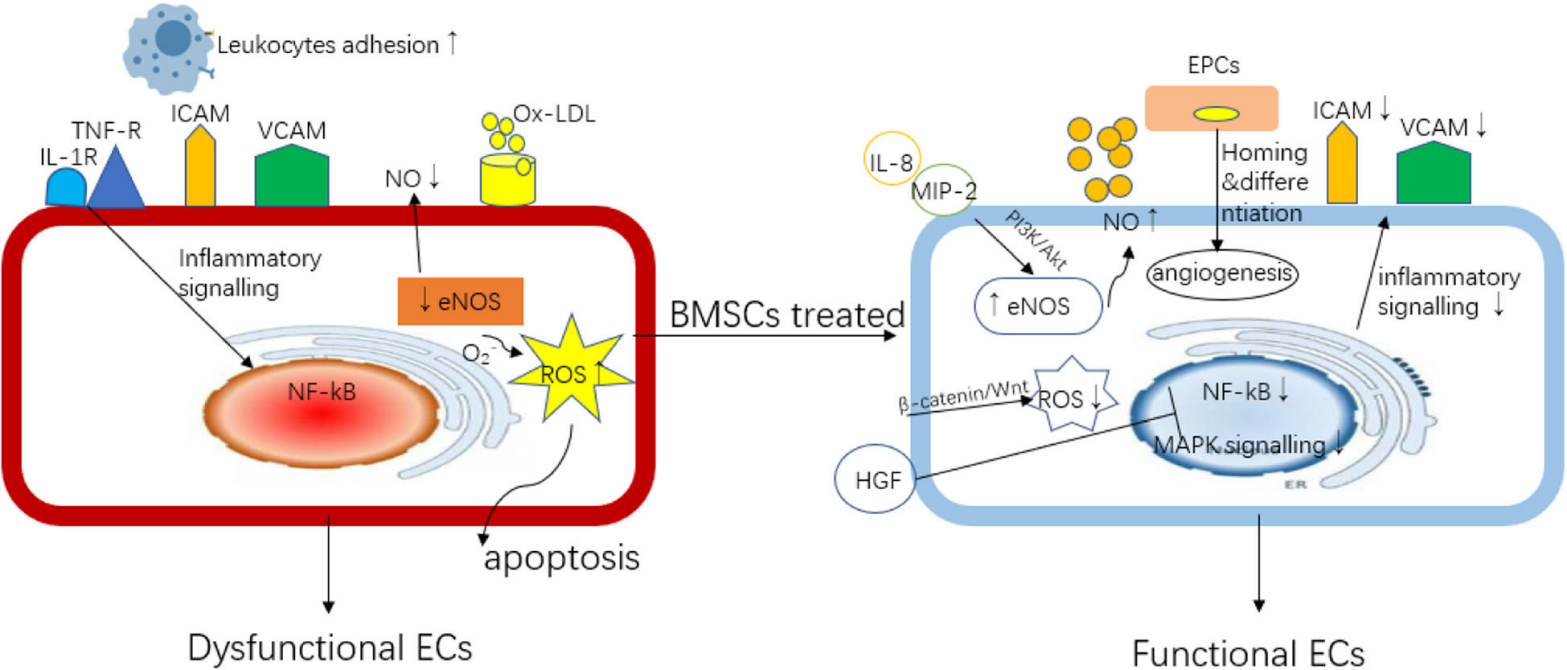
The role of allogeneic BMSCs on ECs

BMSCs may have the capability to regulate the endothelial progenitor cells (EPCs) homing to repair or regulate EC function. The number of EPCs is inversely related to cardiovascular risk, and patients with healthy vessels have a greater number of circulating EPCs [45]. Though it has been found that circulating EPCs rarely contribute to endothelium repairment or regeneration both during the process of plaque development and after plaque rupture in an ApoE^−/−^mice AS model [46], the vasculo-protective role of EPCs, either being bone marrow derived or obtained from local vessel, against atherosclerotic plaque is illustrated in several animal studies [47, 48]. In an in vitro experiment, EPCs isolated from health controls can rescue ECD via vascular regeneration for which a strong paracrine mitogenic effect on mature ECs has been observed [49]. A study has demonstrated that when EPCs and BMSCs were co-cultured, an angiogenesis secretome by BMSCs can confer enhanced proliferation and migration capabilities for EPCs which was associated with better endothelium-restoring function [50]. Transplantation of allogeneic BMSCs into patients with heart failure has resulted in a significant proliferation of functional EPCs and improvement in endothelial function, which in turn restore vascular reactivity [21]. However, limited data has raised some questions. It will be important to distinguish the paracrine secretome secreted by MSCs from EPCs, especially the relative role of MSCs compared with that of endogenous EPCs in treating AS. In other words, it is hard to determine whether EPCs are needed to mediate the therapeutic effects of MSCs or MSCs can directly take the effects on regulating ECD. Due to an absence of clear markers that can define EPCs, there is inevitably an overlapping of EPCs with other types of cells; therefore, it is difficult to exclude all the possibilities that any potential type of cells can also be involved in mediating the therapeutic effects of MSCs. For example, the tie2 promoter has been used extensively to follow the fate of EPCs; however, tie2 is not a specific marker for endothelial lineage and can be expressed in some other nonendothelial cell types, including a fraction of monocytic/macrophage cells [51]. In summary, the protective role of BMSCs on ECD should be carefully studied from various perspectives, with interactions between different types of cells being increasingly recognized along with their direct effects on ECs (Fig. 2).

## The role of allogenic BMSCs on VSMCs

### Phenotypic transition of VSMCs by BMSCs

Vascular smooth muscle cell phenotypic transition plays an important role in the progression and regression of AS. In the pathological environment, a series of alternations in gene expression of VSMCs have led to a transition from contractile phenotype (differentiated) to secretory state (dedifferentiated), manifesting as differences in proliferation, migration, phagocytosis, and secretion of inflammation substances and collagen fibers, all of which can affect the formation and progression of atherosclerotic plaque [52]. In recent years, studies have found that the origins of SMCs in plaques are heterogeneous. Actually, VSMCs may originate from extravascular progenitor cells and myofibroblasts and bone marrow stem cells, and they might well be that these cells are actually the resident cells within the vascular media [53]. However, during the progression of AS, Scar^+^ VSMCs have shown decreases in the expression levels of contractile phenotype marker proteins (such as Myh11, Acta2, and Tagln), and increases in secretory phenotype marker proteins (such as Spp1, Pde1c), which were associated with their increased capability of migration and proliferation [54].

### VSMCs within atherosclerotic plaque by BMSCs

**Fig. 3:**
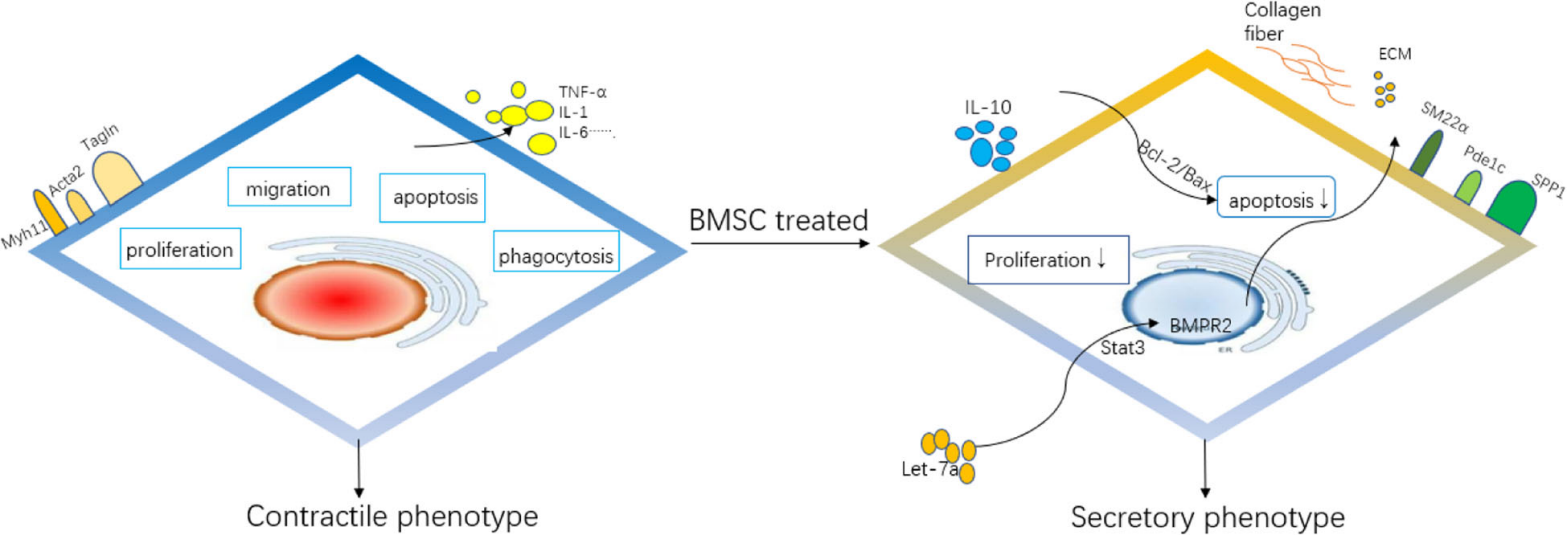
The role of allogeneic BMSCs on VSMCs

It is generally agreed that VSMCs has bidirectional roles in plaque formation. It has been shown that VSMCs secrete inflammatory substances and form foam-like cell in the early phase of plaque formation, whereas in the advanced stage of plaques, VSMCs secrete extracellular matrix proteins and collagen fibers to promote the formation of stable plaques [55]. In fact, BMSCs affect the roles of VSMCs in plaque formation by different regulatory mechanisms. In the early stages of AS, BMSCs inhibit the proliferation of VSMCs by secreting let-7a contained in exosomes and activate the STAT3-BMPR2 signaling pathway [56]. In the advanced plaques in a murine AS model, conditional medium derived from BMSCs scan inhibit the expression of calcification genes such as bone morphogenetic protein 2 (Bmp2), Runt-related transcription factor 2 (Runx2), and homeobox gene 2 (Msx2), and reduce VSMCs apoptosis by activating Bcl-2/Bax signals [57]. These mechanisms may promote a stable plaque (Fig. 3).

### VSMCs reprogramming by BMSCs

Besides, a study showed that SMC-specific ablation of TGF-β signaling in Apoe−/−mice fed western diet results in the reprogramming of VSMCs. These reprogrammed VSMCs have acquired MSC-like phenotype which can further transdifferentiate and generate osteoblasts, chondrocytes, adipocytes, and macrophages, resulting in the vascular abnormalities such as calcification and ossification of the aortic wall and aortic aneurysms [58]. This phenotype further suggests a potential association between VSMCs and BMSCs. However, the mechanism by which BMSCs influence the AS process by regulating VSMC phenotypic changes is not fully understood, and further research is urgently needed.

## The role of BMSCs on macrophages

### BMSCs affect macrophages polarization

**Fig. 4:**
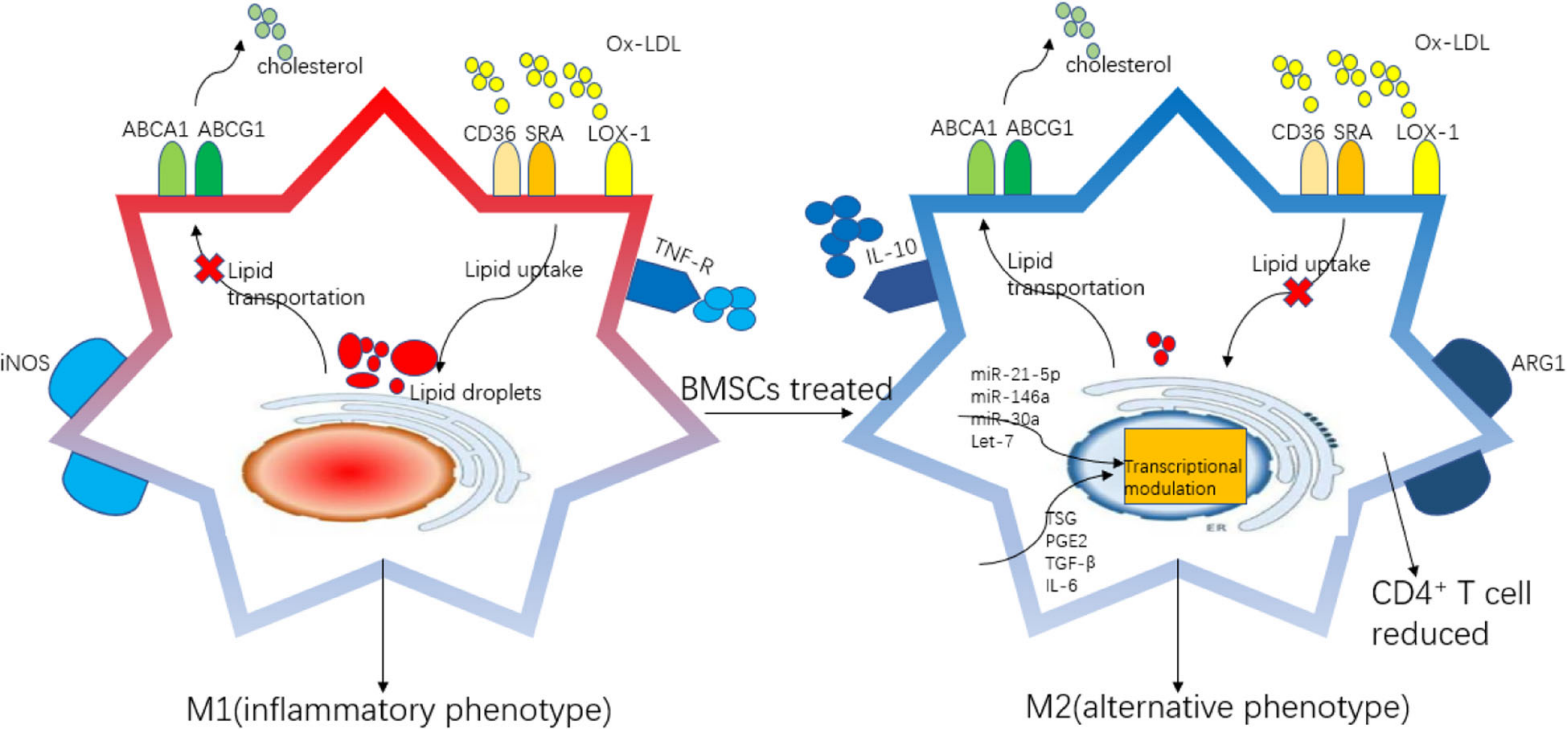
The role of allogeneic BMSCs on macrophages

Macrophages is a key cell type that regulates the innate immune response during the development of AS. Bone marrow-derived monocytes recruit to the circulation and inflamed intima, which they differentiate into macrophages and dendritic cells (DCs) under the sustained stimulation of chronic inflammation, participating in a series of processes such as antigen presentation, lipid phagocytosis, secretion of inflammatory factors, formation of foam-like cells, and in the worst case becoming apoptotic and/or necrotic cells in the development of AS [59]. It is well known that BMSCs can regulate macrophage phenotype polarization, showing a transition from a pro-inflammatory phenotype (M1) to an anti-inflammatory phenotype (M2) (Fig. 4). Different macrophage phenotypes display distinct functions, including phagocytic capacity, secretion profile, antigen presentation ability, proliferation and migration ability, and lipid metabolism ability [60]. It is currently believed that increasing the ratio of M2 and M1 in the plaques can significantly inhibit the progression of plaque. Compared with the control group where atherosclerotic mice models were fed with western diet, atherosclerotic plaque size and lipid deposition were significantly reduced in mice after BMSC transplantation. Moreover, the number of activated macrophages with inflammatory phenotype and macrophage-derived foam cells was all reduced [61].

### BMSCs affect macrophage cholesterol influx and efflux

After co-culture of monocytes with BMSCs, one study showed that the expression of macrophage surface protein arginase 1 (Arg1) was upregulated whereas inducible nitric oxide synthase 2 (iNOS) expression was downregulated; the secretion of inflammatory factors such as TNF-α was reduced in the cell culture supernatant where the anti-inflammatory factors such as IL-10 increased. Moreover, the foam cells decreased significantly after stimulation with oxidized LDL with the expression of lipid phagocytosis receptors Cd36 and Sra1 being downregulated, and the lipid transport receptors ATP binding cassette subfamily A member 1 (Abca1) and ATP binding cassette subfamily G member 1 (Abcg1) were upregulated [26, 62]. Further, an in vivo study showed that BMSCs can deliver a variety of fluorescence-tagged microRNAs to the exosome and were eventually transferred into the macrophages within the plaque, and this can modulate the transcription profile of macrophages in vivo and hence affect or convert the phenotypic function of macrophages. MSC-secreted exosomal miRNAs, which have been reported to regulate macrophage function, include miR-21-5p [63], miR-146a [64], miR-30a [65], and let-7 [66]. In addition, BMSCs simultaneously secrete small molecule soluble substances such as TNF-stimulated gene (TSG), PGE2, TGF-β, and IL-6 to regulate the function of macrophages [67, 68]. Furthermore, the alternated differentiated macrophages within the atherosclerotic lesion may have a role in educating CD4^+^T cell, which also provides a novel insight into the crosstalk between differentiated macrophage and T cells [69]. Of note, according to the process of angiogenesis in mammalian embryos, macrophages, as one of erythro-myeloid progenitors, have the potential of differentiating into EPCs, though the regulation mechanism has not been recognized [70]. Therefore, it will be an interesting question to answer whether BMSCs can influence the impaired endothelial lining through affecting the activity of macrophages.

## The role of BMSCs on T lymphocytes

### BMSCs affect Tregs differentiation

**Fig. 5:**
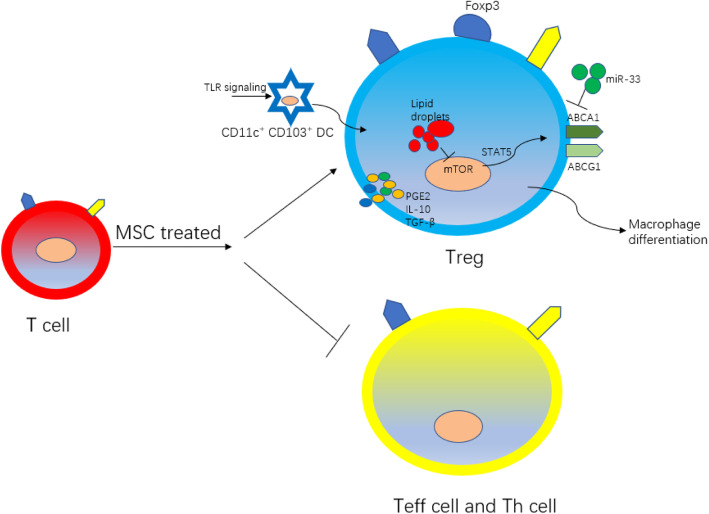
The role of allogeneic BMSCs on T lymphocytes

T lymphocytes are the main cell type that regulates adaptive immune response in AS. Studies have shown that mouse atherosclerotic plaques are predominated by CD4^+^ T lymphocytes, while human atherosclerotic plaques have both CD4^+^ and CD8^+^ T lymphocytes. T lymphocytes can be divided into effector T lymphocytes (Teff cells) and Tregs according to their effects on AS. In the development of AS, phenotypic plasticity can be found to be gradually reduced, with the number of Treg cells reduced and Teff increased [71]. Therefore, we believe that a decrease in the ratio of Tregs to Teff lymphocytes can accelerate plaque progression (Fig. 5). Tregs can directly exert immunosuppressive effects to reduce the overt immune response in plaques and can also indirectly affect the phenotypic differentiation of macrophages to jointly play the role of athero-protection [72]. Current research has found that BMSCs can secrete IL-10, TGF-β, and PGE2 to promote the Treg differentiation by upregulating Foxp3 on the T lymphocyte surface, resulting in a favorable profile of immune components in plaques [73].

### BMSCs affect Tregs differentiation via regulating DCs

It is generally agreed that monocytes recruit to atherosclerotic plaque and differentiate into macrophages and DCs, being involved in the plaque formation. Interestingly, mature DCs that expressed CD11c^+^ CD103^+^ within the plaque have the capability of modulating Treg differentiation [74]. A in vivo murine AS model has shown that BMSC therapy can promote the maturation of DCs within AS lesion, thereby increasing the number of Treg cells herein [75].

### BMSCs affect the crosstalk between macrophages and Tregs

Besides, BMSCs exosomal miR-133 can downregulate the macrophage surface cholesterol efflux-related genes such as ABCA1 and ABCG1, resulting in an interrupted cholesterol efflux and inhibiting foam cell formation. Moreover, these changes are accompanied by a decrease in the number of athero-protective Tregs within the AS lesions, suggesting a potential link between immunometabolism in macrophages and induction of Tregs in the setting of AS pathological process [76]. However, the exact causative relationship between BMSC-mediated macrophage metabolism and Treg induction still remains to be determined. At least, this set of data suggests that both Tregs and macrophage play important roles in BMSC-based therapy for treating AS.

## The role of BMSCs on fibroblasts

In addition to the aforementioned endogenous progenitor cells, fibroblasts are the predominant resident population of the adventitia and responsible for depositing abundant collagen fibrils around vessels [77]. Though few studies have focused on the embryonic origin of these cells, it is generally agreed that they are derived from local mesenchymal cell populations [78, 79]. While fibroblasts exhibit the physiological roles in the static conditions, however, they can differentiate into myofibroblasts when the micro-circumstance of vascular wall has been changed [80]. These activated adventitial myofibroblasts participate in the process of blood vessel remodeling via proliferating, depositing ECM, and secreting inflammatory cytokines and chemokines [81]. In the advanced stage of an atherosclerotic plaque, while media-derived VSMCs predominate in the neointima, the activated adventitial myofibroblasts can also infiltrate the lesion and contribute to the formation of both neointima and fibrous cap through producing a majority of the matrix components [34]. Up to now, there are a lot of questions remained to be answered about the detailed mechanisms by which fibroblasts are involved in the pathological process of vascular abnormalities. This is mainly due to significantly altered gene expression, most of which are shared by many other cell types in vivo, such as VSMCs, MSCs, and ECs [82]. Considering BMSCs have the capability of differentiating into fibroblasts [83], it would be interesting to further investigate how this transdifferentiation would affect the pathological process of atherosclerosis. It certainly warrants further studies to investigate whether the cross-talks between fibroblast with other different types of cells exist, especially with the transplanted BMSCs that have been homing to the AS lesion sites.

## The factors involved in therapeutic effect of MSCs transplantation

### Tissue microenvironment and application routes as decisive factors for MSCs therapy

Emerging evidence have shown that the spectrum of the key components within the secretome from BMSCs is mainly determined by the status of BMSCs which is closely related to their microenvironment. Pathological abnormalities such as calcifications and ossifications can be observed in nearly half of the MI mice receiving BMSCs therapy compared with those with purified hematopoietic progenitor cells, indicating potential deleterious effects of BMSC-based therapy [84]. Even though BMSCs usually only offer a transient paracrine effect rather than survive for a long period and exhibit further differentiation showing aforementioned phenomenon, it remains to be determined whether calcification abnormality in MI heart with BMSCs transplantation is due to the inflammatory environment or the inherent biological fate of BMSCs. Nevertheless, this study has raised an important issue, i.e., the environment has to be taken into account when BMSC-based therapy is instituted. For example, in the tumor environment, MSCs could also exhibit a pro-angiogenic phenotype which promotes the development of tumor [85], while in other inflammatory status, for example, various BMSC-based therapies would achieve immunomodulatory effects as shown in the condition of human umbilical cords (UCMSCs) primed with inflammatory cytokines in the setting of chronic liver disease [86]. For example, in an LPS-induced acute inflammatory mouse model, BMSC transplantation has shown to significantly alleviate the inflammatory response in both pulmonary and circulation. In contrast, BMSC-based therapy showed no effect on the AS mouse models fed with western diet 20 weeks, which is regarded as a chronic inflammatory status in mouse [42]. In addition, the study also reported that application routes of BMSCs are also an important factor that influences BMSC therapeutic effect, including treated period, the transplanted number and method, and the extraction purity of BMSCs. We certainly would assume that a broad range of phenomenon occur when BMSCs therapies are applied to different diseases, offering either beneficial or unfavorable effects.

### Homing as a key step for BMSC-based therapy

Though the crucial role of BMSCs paracrine function has been increasingly recognized, it remains not fully understood how and to what extent the transplanted BMSCs can transit from circulation to the targeted lesions with an impaired vascular wall, as this is actually an initial step, yet a key step, for BMSC-based therapies in atherosclerotic plaque. Mobilization of BMSCs can be mediated by chemokines and inflammatory cytokines, either produced by inflammation cells or other activated BMSCs [87]. Among them, stromal cell-derived factor 1 (SDF-1), recruiting BMSCs from their origin such as bone marrow, and their receptor CXC chemokine receptor 4 (CXCR4), for BMSCs being localized to its target tissue, are the main molecules that mediate the homing process of BMSCs. Blocking SDF-1/CXCR4 signal pathways can result in a remarkable decrease in the number of transplanted stem cells that were recruited to sites of target tissues [88]. It has been shown that multiple pro-homing factors including SDF-1, VEGF-A, and FGF-2 are significantly increased in their plasma level a week after the occurrence of MI in ST elevation myocardial infarction (STEMI) patients, which suggests that the optimal time of homing for stem cell transplantation [89]. However, the detailed underlying mechanisms by which the capability of BMSCs homing to target tissues is enhanced are still not fully understood, which certainly merits further research.

### Origins and sources of MSCs on their therapeutic effects

In addition to the aforementioned findings, BMSCs derived from elderly subjects or patients with peripheral vascular disease showed a decrease in immunomodulation capabilities compared to BMSCs isolated from healthy donors, indicating that the different sources of BMSCs can influence the therapeutic effects of BMSCs. One of the advantages of MSCs is their rich origins, as MSCs can be isolated from various tissues, such as adipocytes, skin, bone marrow, placenta, and umbilical cord. Recent reports have found that MSCs derived from different sources exhibit distinct characteristics in their morphology, the success rate of isolation, colony formation frequency, maintenance period in cell culture, proliferation, and multiple differentiation capacity as well as their immunomodulatory effects [90] (Table 1). An in vitro study found that adipose-derived mesenchymal stem cells (ADSCs) when co-cultured with M1 macrophages exert more advantageous effects over BMSCs, conferring higher anti-inflammatory, phagocytic, anti-apoptotic effects and exhibiting much more enhanced cell viability [91]. In contrast, another study has reported that BMSCs could significantly decrease cytotoxic activity of natural killer cells (NK cells) and increase IFN-γ secretion compared to ADSCs, although both have the capability of inhibiting NK cells proliferation [92]. However, few studies have side-by-side compared the anti-inflammatory and immunomodulation effects of MSCs derived from different origins or sources (i.e., various tissues), especially on the atherosclerotic plaque. The answers to these questions in the future studies would certainly further elucidate the underlying mechanisms by which MSCs exert therapeutic effects on AS.

**Table 1:** Comparative analysis of MSCs from bone marrow, umbilical cord blood, or adipose tissue

Characteristics	BMSCs	ADSCs	UCMSCs
The success rate of isolating	High	High	Low
Colony frequency	Middle	Highest	Lowest
Culture period	Shortest	Middle	Longest
Proliferation capability	Lowest	Middle	Highest
Multiple differentiation capacity	Tri-lineage differentiation	Tri-lineage differentiation	No adipogenic differentiation
Without differences	Morphology, immune phenotype (surface protein expression)		

## Conclusions and perspectives

MSCs, especially the BMSCs obtained from healthy donors, hold great potential for treating patients with AS for which the paracrine effects play the major roles; however, there also exist many factors which can potentially affect the biological functions of BMSCs, and these issues certainly need to be further clarified to facilitate their future clinical applications.

Specifically, some essential aspects related to BMSC-based therapy warrant further investigations. First, due to the rich sources of MSCs, their different origin, probably different environment where the MSCs were isolated, will affect their immune- and inflammatory modulation capability. Data is lacking, especially side-by-side comparisons which should be made between MSCs obtained from these different sources when they are applied to treat patients with AS. Second, a different strategy would be carefully considered when BMSCs therapy was instituted for patients with stable or unstable AS, where the environment for transplanted BMSCs would be totally different, and the data of related basic research for this scenario is much lacking; therefore, much more carefully designed research needs to be carried out to better understand how the different inflammatory stages dictate the final result of BMSC-based therapy. Third, even though the paracrine effects have been regarded as the main underlying mechanism by which BMSCs mediate the inflammatory or immunomodulation, the specific molecule, such as peptides, small non-coding RNA, or other biological active products, should be identified, which would provide a novel therapeutic approach. Lastly, almost no data is available regarding whether and how the transplanted BMSCs are actually recruited to the targeted AS plaque; the detailed mechanism for BMSCs homing should be elucidated which would enhance BMSC-based therapeutic efficacy. Alternatively, the endogenous MSCs can also be re-activated by these fresh energized BMSCs from healthy donors which is probably another interesting mechanism involved in BMSCs therapy. The answers for all the research questions would certainly elucidate some fundamental scientific questions in this important research area and pave the way for wide clinical application for BMSCs therapy in treating patients with AS.

## Data Availability

Not applicable.

## Abbreviations

AS: Atherosclerosis
MSCs: Mesenchymal stem cells
BMSCs: Bone marrow-derived MSCs, adipose-derived MSCs
UCMSCs: Human umbilical cord-derived MSCs
SDF-1: Stromal cell-derived factor 1
CXCR4: CXC chemokine receptor 4
MI: Myocardial infarction
TGF-β: Transforming growth factor beta
VEGF: Vascular endothelial growth factor
HGF: Hepatocyte growth factor
PGE2: Prostaglandin E2
IDO: Indoleamine-2,3-dioxygenase
IGF: Insulin-like growth factor
IL: Interleukin
LPS: Lipopolysaccharide
Tregs: Regulatory T cells
Teff cells: Effector T lymphocytes
TNF-α: Tumor necrosis factor alpha
TSG: TNF-stimulated gene
EVs: Extracellular vesicles
NO: Nitric oxide
VSMCs: Vascular smooth muscle cells
ECs: Endothelial cells
eNOS: Endothelial nitric oxide synthase
ROS: Reactive oxygen species
MIP-2: Macrophage inflammatory protein 2
CAMs: Cell adhesion molecules
MAPK: Mitogen-activated protein kinase
EPCs: Endothelial progenitor cells
NF-kB: Nuclear factor kappa-B
Bmp2: Bone morphogenetic protein 2
Runx2: Runt-related transcription factor 2
Msx2: Homeobox gene 2
Abca1: ATP binding cassette subfamily A number 1
Abcg1: ATP binding cassette subfamily G number 1
Th cells: T helper cells
